# A Molecular Modeling Case Study on the Thermodynamic Partition of DIPNs Derived from Naphthalene and C_3_-Sources Using Non-Shape-Selective Acid Catalysts

**DOI:** 10.3390/molecules30173606

**Published:** 2025-09-03

**Authors:** Wim Buijs

**Affiliations:** Independent Researcher, Lochlehn 237, 6105 Leutasch, Austria; wbuijsm@gmail.com

**Keywords:** isomers, conformers, MMFF, cMMFF, DFT, MP2

## Abstract

Recently, an article was published in which a more accurate pre-screening method compared to MMFF for conformer distributions in flexible organic molecules was presented. However, experimental data on conformer distributions are almost completely lacking. Therefore, old experimental and computational work on the thermodynamic partition of DIPN isomers was revisited to compare the new method, corrected MMFF (cMMFF), with MMFF as a pre-screening tool. Next, the preliminary conformer distributions were used as input for higher-level QM calculations to yield more reliable conformer distributions. Generally, it was found that cMMFF yields smaller energy differences between DIPN isomers and conformers of a DIPN isomer than MMFF, in line with the results of DFT methods B3LYP and B3PW91, used in higher-level calculations. MP2 turned out to be a remarkable outlier, yielding even smaller energy differences both between DIPN isomers and conformers of a DIPN isomer. Preliminary conformer distributions of DIPN isomers obtained with MMFF and optimized with B3LYP and B3PW91 yielded excellent thermodynamic equilibrium partitions of DIPN isomers compared to the available experimental data. Preliminary conformer distributions of DIPN isomers obtained with cMMFF and optimized with B3LYP and B3PW91 performed less well. However, this seems due to a small effect on the energy (~4 kJ/mol) of the local geometry of the β-isopropyl group on naphthalene, which still strongly affects the thermodynamic equilibrium partitions. To obtain a more balanced judgement on the newly proposed method and the existing ones, more comparisons between experimental and computational data on small molecules with a higher degree of flexibility are needed.

## 1. Introduction

Recently, an article was published in which a more accurate estimate of energy differences of conformers in flexible organic molecules compared to MMFF was presented [1]. The method, cMMFF, a so-called correction to the old MMFF model [2,3,4,5,6], is based on a neural network that was trained to reproduce conformer energy differences from ωB97X-V/6-311+G(2df,2p)[6-311G*]//MMFF calculations. According to the authors, cMMFF should not be used to calculate reliable conformer distributions, but as a fast screening to limit the number of conformers that should be considered in higher-level calculations. As experimental data on conformer energy differences are very scarce, the conformer distributions obtained with the high-level method CCSD(T), the “Gold Standard”, are often taken as a reference [7]. Unfortunately, its application is limited due to the high computational time it takes to calculate accurate conformer energy differences of relatively small organic molecules. The need for more experimental data remains undiminished. Therefore, the results of an old research topic were re-examined.

Between 2003 and 2008, a research collaboration between several Belgian universities and Delft University was set up with the primary goal to design catalysts combining high reaction rates and high selectivity via controlled synthesis of zeolitic catalysts with macropores to allow fast transport and micropores for shape-selective transformations [8,9]. In order to conclude whether a particular catalyst is shape-selective, it was necessary to also perform experiments that yield a thermodynamic mixture of diisopropylnaphthalenes (DIPNs). As a promising model system, diisopropylation of naphthalenes to 2,6-diisopropylnaphthalene (2,6-DIPN) with a C_3_ source was chosen because shape selectivity was reported for this system using H-MOR as a catalyst. Initially, no shape selectivity with H-MOR could be experimentally observed. This led to a scientific debate that was eventually resolved by a joint publication by Brzozowski and Buijs [10] in which it was concluded beyond any doubt that shape-selective formation of 2,6-DIPN occurs in the presence of high silica H-MOR with propylene as alkylating agent.

Apart from the experimental results, molecular modeling results played a significant role too in that discussion because the thermodynamic partition of the DIPN isomers was quite successfully determined with several computational methods and compared with the experimentally observed results with non-shape-selective catalysts. In the old research, routinely, MMFF conformer distribution was determined prior to higher-level methods using the lowest energy conformer of a DIPN isomer.

The goal of this article is to compare cMMFF with MMFF as a pre-screening tool before applying the higher-level methods described in the older research.

**Figure 1:**
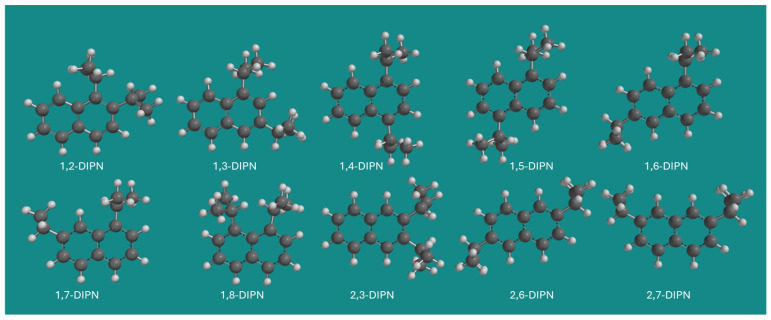
The 10 isomeric DIPNs. Method: MMFF; display: ball and spoke; C: black, H: white.

DIPNs are well-known commodity organic chemicals. A mixture of DIPNs has found application as a solvent for carbonless copy paper. The mixture is also known as Kureha Micro Capsule Oil; however, it is not clear if Kureha still produces the mixture [11,12]. DIPNs can be produced by diisopropylation of naphthalenes using a wide variety of acid catalysts [13]. Ten isomeric DIPN’s are possible: 1,2-DIPN, 1,3-DIPN, 1,4-DIPN, 1,5-DIPN, 1,6-DIPN, 1,7-DIPN, 1,8-DIPN, 2,3-DIPN, 2,6-DIPN, and 2,7-DIPN. Figure 1 provides an overview of all 10 DIPN isomers.

Each DIPN isomer can only have four conformers, which in cases with internal symmetry degenerate to three conformers (1,4- and 1,5-DIPN) or even two conformers (2,6- and 2,7-DIPN). 2,3- and 1,8-DIPN show only two conformers due to the strong steric hindrance of the two neighboring isopropyl groups.

Before discussing the computational results, attention will be paid to the above-mentioned experimental data and how they were obtained. The experimental data themselves will be listed under Section 2.

Diisopropylation of naphthalenes was carried out by Bouvier et al. [8] and Buijs et al. [9] using a 50 mL stainless steel Parr autoclave wherein 320 mg of naphthalene (2.5 mmol), 0.38 mL of isopropanol (5.0 mmol), and 500 mg of a non-shape-selective zeolite catalyst (H-USY) were added to 25 mL of cyclohexane. Reactions were conducted at 200 °C and 24 h at autogenic pressure; however, equilibrium conditions were usually obtained after 4 h. Three aspects should be kept in mind in this approach:

(a) naphthalene conversion (200 °C, 4 h): 91.0%; yield to IPNs: 37.5%, to DIPNs: 38.4%, to PIPNs: 5.0%, to Unsaturates: 4.3% and to Others: 5.8%,
(b) the use of cyclohexane as a solvent,
(c) the stoichiometric ratio of naphthalene/isopropanol = 1/2.

“Unsaturates” are C_3_-oligomerization products, and “Others” consist of cyclohexane-derived byproducts.

Brzozowski and Buijs [10] applied a different experimental procedure. Diisopropylation of naphthalenes was conducted using a 250 cm^3^ stainless steel autoclave wherein 100 g of naphthalene and 5 g of a non-shape-selective Amorphous Silica–Alumina (ASA) catalyst were added. After sealing and inertization of the autoclave, the mixture was heated to the desired reaction temperature, and next, propene was supplied at 8 bar. Reactions were conducted at 250 °C for 6 h.

So, in this case:

(a) naphthalene conversion (250 °C, 4 h): 90.5%; yield to IPNs: 29.5%, to DIPNs: 46.4%, to PIPNs: 19.5%, and to Unsaturates: 4.7%.
(b) no additional solvent,
(c) a constant supply of propene.

The two different experimental procedures lead to slightly different “pseudo” thermodynamic partition of DIPNs. Not only is the total yield to DIPNs different at the same naphthalene conversion but also the amount of PIPNs and other side products. This is mainly due to the C_3_ source, amount, and the way it is added; the catalysts (H-USY, ASA); and the differences in process conditions (temperature and pressure). It should be kept in mind that the thermodynamic partition of DIPNs is merely a theoretical concept, as the experimental systems at least consist of naphthalene, IPNs, DIPNs PIPNs, C_3_-oligomerization products, and solvent-related side products.

## 2. Results

### 2.1. Relative Energies and Structural Properties of DIPN Isomers and Their Conformers

Table 1 provides an overview of the relative energies of the DIPN isomers for the computational methods applied. The first column (Isomer) lists the 10 DIPN isomers.

The second column (c) lists the number of conformers for each DIPN isomer, which varies from two to four. For each isopropyl group, two geometric positions are available in principle, leading to four conformers for each DIPN isomer. Depending on the exact symmetry of the DIPN isomer, it degenerates to either two or three conformers.

The next five columns list the computational methods applied: MMFF, cMMFF, B3LYP/6-31G*, B3PW91/6-31G*, and MP2/6-31G*. CMMFF (corrected MMFF) is the neural network option based on ωB97X-V/6-311+G(2df,2p)[6-311G*]//MMFF calculations, as described by Hehre et al. [1]. The QM codes were used in the older research [9,10].

Each of these columns is divided into two sub-columns displaying the relative energy to the lowest energy DIPN isomer (rel. E lei), and the relative energy to its corresponding lowest energy conformer (rel. E lec), both following the order of conformers according to MMFF, as displayed in columns 2 and 3.

Generally, DFT computational results, including cMMFF, lead to smaller energy differences between DIPN isomers and between conformers of a DIPN isomer, compared to MMFF molecular mechanics. Of course, this was expected by the very nature of the MM versus the QM methods.

In four cases, 1,2-DIPN, 1,3-DIPN, 1,4-DIPN, and 1,5-DIPN, the order for all conformers of each DIPN isomer is consistent across all computational methods. All conformers show the isopropyl C-H almost in the naphthalene plane. No conformers are observed with an isopropyl CH_3_ group close to the naphthalene plane.

In four cases, 1,6-DIPN, 1,7-DIPN, 2,6-DIPN, and 2,7-DIPN, the order for all conformers is consistent across cMMFF and the DFT methods but not across MMFF. In two cases, 1,6-DIPN and 1,7-DIPN, the original MMFF conformer 2 is the lowest energy conformer according to cMMFF, the DFT methods, and MP2. In the other two cases, 2,6-DIPN and 2,7-DIPN, the original MMFF conformer 3 is the lowest energy conformer according to cMMFF, the DFT methods, and MP2.

The differences between MMFF on one side and cMMFF, B3LYP/6-31G*, B3PW91/6-31G*, and MP2/6-31G* on the other side are caused by the method-dependent quantitative effect on the energy of the local geometry of the β-isopropyl group on naphthalene. 1,6-DIPN and 1,7-DIPN have one β-isopropyl group, while 2,6-DIPN and 2,7-DIPN have two β-isopropyl groups. The effect is discussed for 2,6-DIPN.

**Figure 2:**
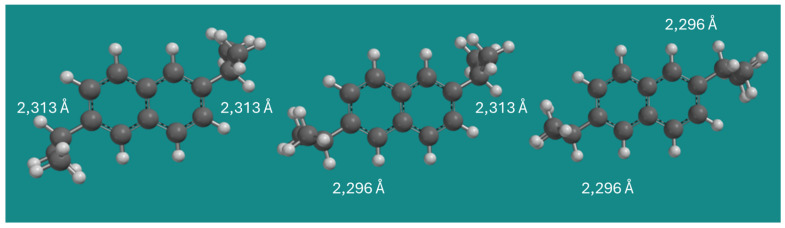
Conformers of 2,6-DIPN. Method: MMFF; display: ball and spoke; C: black, H: white.

Figure 2 shows the three conformers of 2,6-DIPN. As can be seen in Table 1, the strain energy difference between conformer 1 and 2 in MMFF is only 0.11 kJ/mol, and the strain energy difference between conformer 1 and 3 of 2,6-DIPN is 0.23 kJ/mol. The effect is additive and extremely small.

In MMFF, H-H distances between an isopropyl C(2)-H and an α- or β-H on naphthalene vary by only ~0.02 Å. In the QM methods applied, all intramolecular H-H distances are 2.291 Å ± 0.002 Å, but the energy differences between conformers 1 and 2, and conformers 1 and 3 are approximately −2.00 and −4.00 kJ/mol, respectively. Again, the effect is additive and small, however, with a major influence on the thermodynamic equilibrium partition. There is no easy explanation for this small discrepancy between MMFF and the QM methods. It seems to be an electronic effect that is not being accounted for in MMFF. The effect is not related to a difference in dispersive interactions: applying B3LYP-D3, a reparameterization of B3LYP by Grimme et al. [14], to provide a better account of dispersive interactions inside and between molecules, leads to an extremely small decrease in relative energy between conformer 1 and 3 of 2,6-DIPN of only 0.03 kJ/mol compared to the original B3LYP: −3.37 vs. −3.40 kJ/mol.

MP2/6-31G* yields even smaller differences between the relative energies of the DIPN isomers but not between the conformers of a DIPN isomer.

### 2.2. Thermodynamic Equilibrium Distribution of DIPN Isomers

Table 2 and Table 3 list the thermodynamic equilibrium partitions experimentally and computationally derived at 200 and 250 °C. The experimental data in Table 2 on the non-shape-selective catalysts H-USY and Zeogrid-50 originate from the same research project on the diisopropylation of naphthalene with isopropanol of Bouvier et al. [8].

The second column (*p*) lists the number of ways a DIPN isomer can be formed. For the correct calculation of the thermodynamic partition of the 10 DIPN isomers, it is important to realize that each of the isomers can be formed in more than one way. As there are four equivalent α and β-positions on naphthalene, each α,β-DIPN can be formed in four ways, while each α,α-DIPN and β,β-DIPN can be formed in two ways, thus, the α,β-DIPN isomer 1,3-DIPN = 2,4-DIPN = 5,7-DIPN = 6,8-DIPN and the α,α-DIPN isomer 1,4-DIPN = 5,8-DIPN, etc.

For the calculation of the thermodynamic equilibrium partition, the relative energies of the DIPN isomers are required, as displayed in Table 1. For the comparison of the pre-screening with MMFF and cMMFF, the lowest energy conformer from MMFF and cMMFF was taken for each DIPN isomer and fully optimized using B3LYP/6-31G*, B3PW91/6-31G*, and MP2/6-31G*. The relative energies were calculated using the lowest energy DIPN isomer, either 2,6-DIPN or 2,7-DIPN.

The thermodynamic equilibrium partition was calculated using the formula below:

$$\%\;DIPN\;isomer\left(n\right)=\frac{p\left(n\right)\times e^{-Erel\;DIPN\;isomer\;(n)/RT}}{\sum p(n)\times e^{-Erel\;DIPN\;isomer\;(n)/RT}}\times 100\%$$

The experimental values obtained with the two catalysts do show some variations. H-USY shows lower values for α,β-DIPNs, 1,3-, 1,6-, and 1,7-DIPN than ZG-50, while the relative amounts of 2,6- and 2,7-DIPN are 36.3 and 42.2% for H-USY and 38.4 and 38.1% for ZG-50. ZG-50, with its large pores to allow fast transport of reactants and products, shows a naphthalene conversion of 96%, while H-USY shows a conversion of 90%. With ZG-50, the yield to IPNs is 20%, to DIPNs 50%, to PIPNs 21%, and to cyclohexane-related side products 5%. With H-USY, the yield to IPNs is 38%, to DIPNs 39%, to PIPNs 6%, and to cyclohexane-related side products 7%. So, the faster catalyst ZG-50 might lead not only to a higher yield of DIPNs but also to a slightly closer approach to the thermodynamic equilibrium of DIPN isomers.

The thermodynamic equilibrium partitions obtained with B3LYP/6-31G* and B3PW91/6-31G* are close to the experimentally obtained partitions, but surprisingly, using the lowest energy conformers from MMFF yields an even better partition than using the lowest energy conformers from cMMFF. The effect is entirely due to the previously discussed effect on the energy of the local geometry of the β-isopropyl group on naphthalene.

The thermodynamic equilibrium partitions obtained with MP2/6-31G* are not as good as the partitions obtained with the two DFT codes. As described above, the differences in relative energies of the DIPN isomers are the lowest of all methods applied, leading to partitions that are overpopulated in α,β-DIPN isomers and underpopulated in β, β-DIPN isomers.

It is worth mentioning that adding thermodynamic enthalpy corrections did not improve the partitions obtained with all QM methods.

The partition of DIPNs obtained with the non-shape-selective catalyst H-USY at 250 °C is, at first sight, contradictory to the results obtained at 200 °C. As explained in the Introduction, the differences in reaction conditions, particularly the constant supply of propene at 250 °C versus the stoichiometric amount of isopropanol (naphthalene/isopropanol = ½), are the main cause of the difference, which generally leads to di- and tri-isopropylated products. Still, H-USY produces slightly more 2,7- than 2,6-DIPN, and the amounts of some α,β-DIPNs differ too. The non-shape-selective catalyst ASA behaves like ZG-50 with respect to the partition of DIPNs and its small preference for 2,6-DIPN.

The thermodynamic equilibrium partitions obtained with B3LYP/6-31G*, B3PW91/6-31G* again coincide very well with the experimental ones, while MP2/6-31G* again is the outlier. The effect of the difference in temperature of 50 °C is rather limited to ~2% each on the amounts of 2,6- and 2,7-DIPN.

## 3. Discussion

This study was initiated by the recent article of Hehre et al. [1] wherein a corrected MMFF molecular mechanics model is presented as a more accurate pre-screening tool for flexible organic molecules to obtain preliminary conformer distributions, which should be further subjected to higher-level QM calculations. Therefore, older experimental and computational work on DIPNs was revisited [8,9,10,15]. As each of the 10 DIPN isomers can only have four conformers, the overall computational effort to yield high-level QM conformer distributions is limited, while at the same time, the thermodynamic equilibrium partition can be calculated, based on the lowest energy conformer obtained with either MMFF or cMMFF, each of them fully optimized on a high QM level. Furthermore, the system profits from the fact that the total energies of DIPN isomers are compared, and these isomers are completely isodesmic, so the inherent errors of the QM codes are largely cancelled.

B3LYP/6-31G* and B3PW91/6-31G* lead to thermodynamic equilibrium partitions of DIPNs, which are in good agreement with the old experimental and computational findings. At least in this case, MMFF performs slightly better than cMMFF in the pre-screening. The difference between the two methods is caused by a small effect of ~2 kJ/mol on the energy of the local geometry of a non-hindered β-isopropyl group on naphthalene. As the effect is cumulative in 2,6- and 2,7-DIPN, the overall effect is ~4 kJ/mol for all QM methods applied, as discussed under 2.1.

MP2/6-31G* is a remarkable outlier. The energy differences between the DIPN isomers are significantly lower, leading to worse thermodynamic equilibrium partitions in MMFF- and cMMFF-based results.

This is one case only, and future case studies, using experimental data on small molecules with higher flexibility, are needed for a final assessment of the proposed new methodology, cMMFF, for rapid pre-screening of conformer distributions.

## 4. Conclusions

1. Thermodynamic equilibrium partitions of DIPN isomers were obtained with B3LYP/6-31G*, B3PW91/6-31G*, and MP2/6-31G* calculations on the lowest energy conformers obtained with MMFF and cMMFF as pre-screening methods.
2. Thermodynamic equilibrium partitions of DIPN isomers obtained with B3LYP/6-31G* and B3PW91/6-31G*, using MMFF as a pre-screening method, agree better with the experimental results obtained earlier than results obtained with cMMFF as a pre-screening method.
3. The observed difference between the two methods is due to an effect of ~2 kJ/mol on the energy of the local geometry of a non-hindered β-isopropyl group on naphthalene.
4. The DIPN case demonstrates what level of computational accuracy is required to obtain reliable thermodynamic equilibrium partitions of isomers.
5. MP2/6-31G* is a remarkable outlier, due to the significantly lower energy differences between the DIPN isomers, leading to worse thermodynamic equilibrium partitions.

## 5. Materials and Methods

Spartan’24 [16] was used for all calculations. This package not only contains many methods, ranging from Molecular Mechanics to Density Functional codes, but also, a series of common tasks are available. The tasks Conformer Distribution and Equilibrium Geometry were used frequently. Furthermore, a wide range of molecular properties can be calculated.

Frequency calculations were made, and thermodynamic (gas phase) enthalpy corrections were applied routinely. Entropy corrections were not made because of the huge simplifications of the actual systems.

The Merck Molecular Force Field (MMFF) [2,3,4,5,6] and the corrected MMFF (cMMFF) [1] were used to obtain primary conformer distributions. Next, these CDs were used as starting structures for quantum chemical CD calculations using various DFT codes. To ease the understanding of the results, the original order of the CDs obtained with MMFF was maintained in the CDs obtained with DFT.

B3LYP [17,18,19,20] and the related B3PW91 [21,22] were used as originally described.

Intrinsic errors in DFT calculations were reduced by applying the isodesmic concept [23], leading to gross cancellation of these errors in total energies. N.B. All DIPN isomers and related conformers are inherently isodesmic.

All molecular structures can be downloaded from the Supplementary Materials as .mol2 files. All calculations are available in Supplementary Materials too as an Excel file. Finally the Guidance Supplementary Materials document provides detailed information on the methods applied and how to restore all properties.

## Figures and Tables

**Table 1:** Relative energies (kJ/mol) of DIPN isomers and conformers applying various computational methods; c: conformer number. rel E lei: relative energy compared to the lowest energy isomer, rel E lec: relative energy compared to the lowest conformer of an isomer.

Isomer	c	MMFF		cMMFF		B3LYP/6-31G^*^		B3PW91/6-31G^*^		MP2/6-31G^*^	
		rel E lei	rel E lec	rel E lei	rel E lec	rel E lei	rel E lec	rel E lei	rel E lec	rel E lei	rel E lec
1,2-DIPN	1	57.99	0.00	41.86	0.00	46.06	0.00	45.80	0.00	36.29	0.0
	2		3.57		0.47		0.99		1.00		1.37
	3		10.78		6.48		7.52		8.14		7.72
	4		47.24		35.31		35.92		36.66		39.21
1,3-DIPN	1	19.99	0.00	12.52	0.00	12.32	0.00	11.91	0.00	6.86	0.00
	2		0.25		2.00		3.78		3.82		5.17
	3		7.48		7.29		6.50		7.25		7.78
	4		7.55		9.04		8.24		8.90		9.67
1,4-DIPN	1	44.36	0.00	27.30	0.00	25.36	0.00	24.43	0.00	16.68	0.00
	2		7.97		7.79		7.23		7.81		8.11
	3		16.29		15.71		14.43		15.85		16.46
1,5-DIPN	1	44.14	0.00	26.11	0.00	25.63	0.00	25.01	0.00	18.02	0.00
	2		8.28		7.79		7.56		8.17		7.83
	3		16.58		15.82		15.90		16.31		16.21
1,6-DIPN	1	20.03	0.00		1.81		0.00		0.00		0.00
	2		0.10	13.37	0.00	12.01	−1.84	11.46	−1.81	7.87	−1.82
	3		7.21		8.75		6.82		7.52		7.45
	4		7.31		6.94		4.99		5.64		2.60
1,7-DIPN	1	19.83	0.00		1.10		0.00		0.00		0.00
	2		0.47	14.57	0.00	12.49	−1.85	11.83	−2.19	7.50	−1.28
	3		6.73		7.86		7.11		7.87		6.68
	4		7.55		7.32		5.17		5.58		6.00
1,8-DIPN	1	82.53	0.00	61.33	0.00	66.52	0.00	65.78	0.00	53.21	0.00
	2		34.58		29.44		25.10		26.07		27.10
2,3-DIPN	1	19.24	0.00	17.23	0.00	19.30	0.00	18.99	0.00	12.69	0.00
	2		9.17		7.11		7.39		8.06		7.99
2,6-DIPN	1	0.05	0.00	0.00	0.00		0.00		0.00		0.00
	2		0.11		−1.93		−1.72		−2.06		−1.90
	3		0.23		−3.85	0.56	−3.40	0.00	−4.05	0.00	−3.72
2,7-DIPN	1	0.00	0.00	0.02	0.00		0.00		0.00		0.00
	2		0.15		−1.93		−2.06		−2.11		−1.91
	3		0.28		−3.83	0.00	−4.04	0.14	−4.08	0.16	−3.73

**Table 2:** Experimental and computational thermodynamic equilibrium partitions of DIPNs at 200 °C using the most abundant conformer according to MMFF or cMMFF as pre-screening; ^a^ non-shape-selective catalyst H-USY [8] p56, ^b^ non-shape-selective catalyst Zeogrid 50 [8] p91.

Isomer	*p*	H-USY ^a^	ZG-50. ^b^	B3LYP/6-31G^*^		B3PW91/6-31G^*^		MP2/6-31G^*^	
				MMFF	cMMFF	MMFF	cMMFF	MMFF	cMMFF
		%							
1,2-DIPN	4	0.0	0.0	0.0	0.0	0.0	0.0	0.0	0.0
1,3-DIPN	4	5.5	8.9	9.4	4.1	10.4	4.2	22.4	11.9
1,4-DIPN	2	0.7	0.0	0.2	0.1	0.2	0.1	0.9	0.5
1,5-DIPN	2	2.0	0.4	0.2	0.1	0.2	0.1	0.7	0.3
1,6-DIPN	4	5.2	7.1	6.3	4.4	7.3	4.8	10.9	9.2
1,7-DIPN	4	4.9	6.4	5.6	3.9	6.1	4.3	13.8	10.1
1,8-DIPN	2	0.0	0.0	0.0	0.0	0.0	0.0	0.0	0.0
2,3-DIPN	2	3.5	0.8	0.8	0.3	0.9	0.4	2.5	1.3
2,6-DIPN	2	36.3	38.4	39.1	40.5	38.3	43.8	24.9	34.0
2,7-DIPN	2	42.2	38.1	38.4	46.7	36.7	42.3	23.9	32.6
Sum		100.0	99.8	100.0	100.0	100.0	100.0	100.0	100.0

**Table 3:** Experimental and computational thermodynamic equilibrium partitions of DIPNs at 250 °C; ^a^ non-shape-selective catalyst H-USY [15], ^b^ non-shape-selective catalyst ASA [10].

Isomer	*p*	H-USY ^a^	ASA. ^b^	B3LYP/6-31G^*^		B3PW91/6-31G^*^		MP2/6-31G^*^	
				MMFF	cMMFF	MMFF	cMMFF	MMFF	cMMFF
		%							
1,2-DIPN	4	0.0	0.0	0.0	0.0	0.0	0.0	0.0	0.0
1,3-DIPN	4	3.8	11.1	10.8	5.2	11.8	5.4	22.7	13.1
1,4-DIPN	2	0.1	0.3	0.3	0.1	0.3	0.2	1.2	0.7
1,5-DIPN	2	0.1	0.2	0.3	0.1	0.3	0.1	0.9	0.5
1,6-DIPN	4	7.5	7.3	7.6	5.6	8.6	6.0	11.8	10.4
1,7-DIPN	4	4.9	5.9	6.8	5.0	7.2	5.5	14.6	11.3
1,8-DIPN	2	0.0	0.0	0.0	0.0	0.0	0.0	0.0	0.0
2,3-DIPN	2	0.4	0.6	1.1	0.5	1.2	0.5	3.0	1.7
2,6-DIPN	2	40.3	38.8	36.9	39.0	36.0	41.8	23.4	31.7
2,7-DIPN	2	42.9	35.6	36.3	44.4	34.6	40.5	22.5	30.6
Sum		100.0	99.8	100.0	100.0	100.0	100.0	100.0	100.0

## Data Availability

All primary molecular structures and secondary calculations are available in the Supplementary Materials (Molecular Structures, Molecules Thermo Partition DIPNs).

## Supplementary Materials

The following Supporting Information can be downloaded at: https://www.mdpi.com/article/10.3390/molecules30173606/s1, Guidance Supplementary Materials (.docx), Molecular structures (.mol2), Molecules Thermo Partition DIPNs (.xslx),

## Abbreviations

The following abbreviations are used in this manuscript:

MMFF	Merck Molecular Force Field [2,3,4,5,6]
cMMFF	Corrected Merck Molecular Force Field [1]
DFT	Density Functional Theory
CD	Conformer Distribution
DIPN	Diisopropylnaphthalene
PIPN	Polyisopropylnaphthalene
H-MOR	High (Temperature) Modulus of Rupture
H-USY	H+-form Ultra Stable Y-type zeolite
ZG-50	Zeolitic catalyst Zeogrid-50 [11] page 80–87
