# Supplementary material for: A Molecular Modeling Case Study on the Thermodynamic Partition of DIPNs Derived from Naphthalene and C3-Sources Using Non-Shape-Selective Acid Catalysts
Source: Molecules. 2025 Sep 3;30(17):3606. doi: 10.3390/molecules30173606 (PMC12430352; doi:10.3390/molecules30173606)
Supplement: Supplementary file 1 [file molecules-30-03606-s001.zip › Guidance Supllementary Materials.pdf]

Guidance Supplementary Materials

A Molecular Modeling Case study to the Thermodynamic Partition of DIPN's derived from Naphthalene and C3-sources using non-shape selective acid catalysts

Wim Buijs* wbuijsm@gmail.com ORCID: orcid.org/0000-0003-3273-5063

Contents

Supplementary Materials contains:

1. Molecules Thermo Partition DIPNs (Excel; xslx).
2. CD MMFF: [.mol2]

Ad 1: The Molecules Thermo Partition DIPNs excel file contains all data and secondary calculations used or briefly mentioned in the article with a short description in the first 2 lines.

Ad 2.: The molecular structures [.mol2] are listed in a folder named CD MMFF mol2. Each entry, like CD 1,2-DIPN_MMFF_Conf.mol2, contains all conformers of the named DIPN isomer.

To restore all quantitative data from the [.mol2] files, a full geometry optimization should be carried out using one of the methods applied in the article, like MMFF, cMMFF, B3LYP/6-31G*, B3PW91/6-31G*, and MP2/6-31G*. All methods yield the same number of conformers for each DIPN isomer. As the geometry of all structures is very similar, this will not take much computational time. Furthermore a frequency calculation should be applied to enable the addition of enthalpy corrections to the total energy.

B3LYP is applied as originally described, using for Exchange: 0.2000 Hartree-Fock + 0.0800 Slater + 0.7200 B88 and for Correlation: 0.1900 VWN1RPA + 0.8100 LYP

General Convergence Criteria:
SCF tolerance = $1*10^{-7}$ Hartree; Geometry optimization Gradient tolerance = $7*10^{-4}$ Hartree/Bohr and the Distance tolerance =$1.4*10^{-3}$ Å

All calculations can in principle be carried out with a variety of (commercial) molecular simulations packages like Spartan, GAUSSIAN, Materials Studio or SCN/AMS.
